# Over a Decade of Maxillofacial PEEK Patient-Specific Innovation: A Retrospective Review of the Evolution from In-House Craft to Virtual Design and Remote Manufacturing

**DOI:** 10.3390/cmtr19010008

**Published:** 2026-01-21

**Authors:** Nicholas J. Lee, Gareth Honeybone, Mohammed Anabtawi, Mathew Thomas, Sachin M. Salvi

**Affiliations:** 1 Sheffield Teaching Hospitals NHS Foundation Trust, Glossop Rd, Broomhall, Sheffield S10 2JF, UK; nicholas.lee@nhs.net (N.J.L.); sachin.salvi@nhs.net (S.M.S.); 2 Leeds Hospitals NHS Trust, Great George St, Leeds LS1 3EX, UK; 3 University Hospitals of Derby and Burton NHS Foundation Trust, Uttoxeter Rd, Derby DE22 3NE, UK

**Keywords:** polyetheretherketone, PEEK, facial deformity, interlocking, onlay, bespoke maxillofacial prosthesis

## Abstract

Maxillofacial skeletal reconstruction presents significant challenges due to anatomical complexity, functional requirements, and aesthetic demands. Traditional materials such as titanium and autogenous bone grafts have limitations, prompting interest in Polyetheretherketone (PEEK), a versatile thermoplastic polymer with advantages like biocompatibility, radiolucency, and elasticity similar to human bone. This multi-year case series evaluates the clinical outcomes of PEEK implants used in 56 cases on 53 patients for maxillofacial reconstruction, primarily for trauma (44 patients) and deformity (9 patients). PEEK implants were applied to various facial regions including the orbit, zygoma, mandible, and maxilla. The majority of surgeries utilised virtual surgical planning. Patient-specific implants were fabricated using 3D imaging technologies, allowing customisation for optimal fit and functionality. The mean patient age was 37 years with a split of 37 to 16 females. Some complications were noted such as infection and paraesthesia. However, the majority of patients experienced positive outcomes. The findings support PEEK implants as a safe, effective, and adaptable material for maxillofacial surgery, with potential for further advancements in material properties and surgical technologies to improve long-term outcomes.

## 1. Introduction

Polyetheretherketone (PEEK) is a semi-crystalline, linear aromatic polymer recognised for its excellent biocompatibility, making it highly suitable for craniofacial reconstruction. It provides several advantages: radiolucency to X-rays, non-magnetic properties (avoiding artefacts on computed tomography [CT] and magnetic resonance [MR] imaging), non-allergenicity, low weight, resistance to fatigue and chemical degradation, and stability of mechanical properties following repeated sterilisation cycles [1].

Intraoperatively, PEEK can be readily contoured using high-speed burs and secured to bone with conventional plating systems. The integration of computer-aided design and computer-aided manufacturing (CAD/CAM) has further improved the accuracy and reproducibility of patient-specific PEEK implants [2,3,4].

Recent studies have identified PEEK as a promising alternative material for maxillofacial reconstruction, owing to its favourable biocompatibility, radiolucency, chemical stability, and elastic modulus, which more closely approximates that of native bone tissue [5].

Craniofacial reconstruction remains challenging due to the intricate anatomy of the facial skeleton, its essential functional roles in mastication and speech, and the high aesthetic demands associated with restoration. Conventional reconstructive approaches have utilised titanium implants, which, while reliable, are limited by their stiffness mismatch with bone and lack of intraoperative adaptability. Similarly, autogenous bone grafts present an additional risk of donor site morbidity and infection.

This study reports on 12 years of clinical outcomes from a single maxillofacial unit, comprising 56 operations on 53 patients who underwent facial reconstruction with patient-specific PEEK implants for both trauma and deformity. In total, 69 implants were planned and 68 successfully placed as some patients required multiple implants during a single operation. The series reflects our progressive experience with PEEK technology, documenting advances in implant design, manufacturing methods, and the integration of navigational systems to optimise intraoperative placement.

## 2. Materials and Methods

A retrospective review of consecutive PEEK implants planned by a single surgeon and placed by the planning surgeon or the planning surgeon and a second surgeon. All implants were placed at the Royal Hallamshire Hospital, Sheffield, UK.

Preoperatively, each patient underwent imaging with either a cone beam computed tomography (CBCT) scan, or a high-resolution multislice computed tomography (CT) scan. For CBCT, a 512 × 512 matrix was used with a maximum slice thickness of 1.0 mm, following a standardised protocol with the patient positioned in natural head position (NHP). NHP provides a reproducible and standardised orientation of the head, defined as the upright posture with the eyes focused on a distant point at eye level, ensuring a horizontal visual axis [6].

We explored an analogue technique for the production of patient-specific PEEK implants, developed locally through the Sheffield Anaplastology Laboratory. The process began with acquisition of high-resolution CBCT/CT data (0.625 mm slices), which were used to generate a stereolithographic skull model. The area of the proposed reconstruction was outlined on the model, and a patient-specific wax template was sculpted directly onto the surface. This wax pattern was then converted into an acrylic implant using a two-part silicone mould and auto-polymerising polymethyl methacrylate, polymerised under pressure for dimensional stability. The resulting acrylic pattern was carefully checked for accuracy and subsequently scanned with a laser scanner to generate a stereolithographic (.stl) file. This digital file was forwarded to a manufacturing facility in Hull, where it was used to direct computer numerical control (CNC) milling of the prosthesis from implant-grade PEEK (Medipeek IM). Only minimal peripheral finishing was required before sterilisation in an autoclave and surgical implantation. We refer to this process as Digital–Analogue PEEK, a hybrid workflow combining traditional analogue modelling with digital scanning and CNC milling. Although this technique involved multiple manual steps and occasional minor adjustments for fit, it was used successfully in a series of patients, including zygomatic reconstructions, and provided a cost-effective means of delivering patient-specific PEEK implants.

The majority of our implants were planned virtually via a planning webinars performed with the surgeon and the clinical engineer to allow computer-aided design (CAD) of implants, with attention to the path of insertion and the avoidance of anatomical obstacles.

Computer-aided manufacturing (CAM) and 3D milling allowed the production of bespoke implants with complex morphology by the manufacturer (DePuy Synthes, Oberdorf, Switzerland).

Before insertion, all patients were reviewed with their 3D planning report, and informed consent for placement of their implant was obtained.

All patients were warned of risks and complications, including infection, residual asymmetry, extrusion, trigeminal nerve injuries, and, for mandibular implants, facial nerve palsy. Smokers were also signposted to further smoking cessation advice.

All surgeries were completed under general anaesthesia with one dose of intravenous antibiotics preoperatively. All zygoma implants were placed via an intra-oral approach; most orbital implants were placed via a transconjunctival approach with or without other additional surgical incision extensions such as lower eyelid swing, lateral canthotomy, or retrocaruncular incision.

A mucoperiosteal flap was designed and performed to achieve a useful subperiosteally pocket without breaching the periosteum.

All implants were soaked with Gentamicin before insertion.

To prevent rotation, implants were fixed with two, Matrix Mid-face or Mandible titanium, screws (DePuy Synthes). If the contour of the implant prevented rotation, then only one screw would suffice. For multiple segments, once interlocked together, two screws were often only required to stabilise the multiple segments now interlocked as a single implant.

Meticulous tension-free layered closure was applied to decrease the risk of wound break down, implant contamination, and exposure. Patients were discharged home with advice on oral hygiene, a soft diet, regular chlorhexidine mouthwashes, and 5 days postoperative oral antibiotics.

The following information was recorded:

- Patient details (demographics, smoking, relevant medications and medical history);
- Type of deformity;
- Side and site of implant;
- Number of segments and number of fixation screws;
- Surgical approach;
- Complications including infection, implant exposure/migration, implant removal, wound dehiscence, and implant-related facial paraesthesia;
- Follow up period.

## 3. Results

The total number of patients treated was 53. The total number of operations was 56 as three patients required a second surgery. The mean age of patients at the date of surgery was 37 years, with an age range from 17 to 73 years, indicating the applicability of PEEK implants across different age groups. The higher proportion of male patients (37 males vs. 16 females) may reflect a gender bias in the incidence of facial trauma or the likelihood of undergoing reconstructive surgery. The patient population was generally healthy, with 36 non-smokers vs. 17 smokers and 39 individuals without significant comorbidities. This demographic profile suggests that the outcomes reported may be more favourable than in a less healthy population, yet it provides a solid baseline for assessing the potential of PEEK implants.

Trauma was the primary indication for the use of PEEK implants, accounting for 44 out of 53 cases, compared to 9 cases due to deformities. The zygoma (15 cases) and orbital (29 cases) regions were the most frequently affected sites with 4 cases requiring both orbital and zygoma implants, reflecting the common involvement of these areas in facial injuries. The remaining cases involved the mandible (6 cases) and maxilla (1 case).

The four cases required a two-part orbito-zygomatic implant. This was made of two segments placed at right angles to each other. The inferior segment was inserted via an intra-oral approach and the superior segment via trans-orbital incisions.

**Figure 1:**
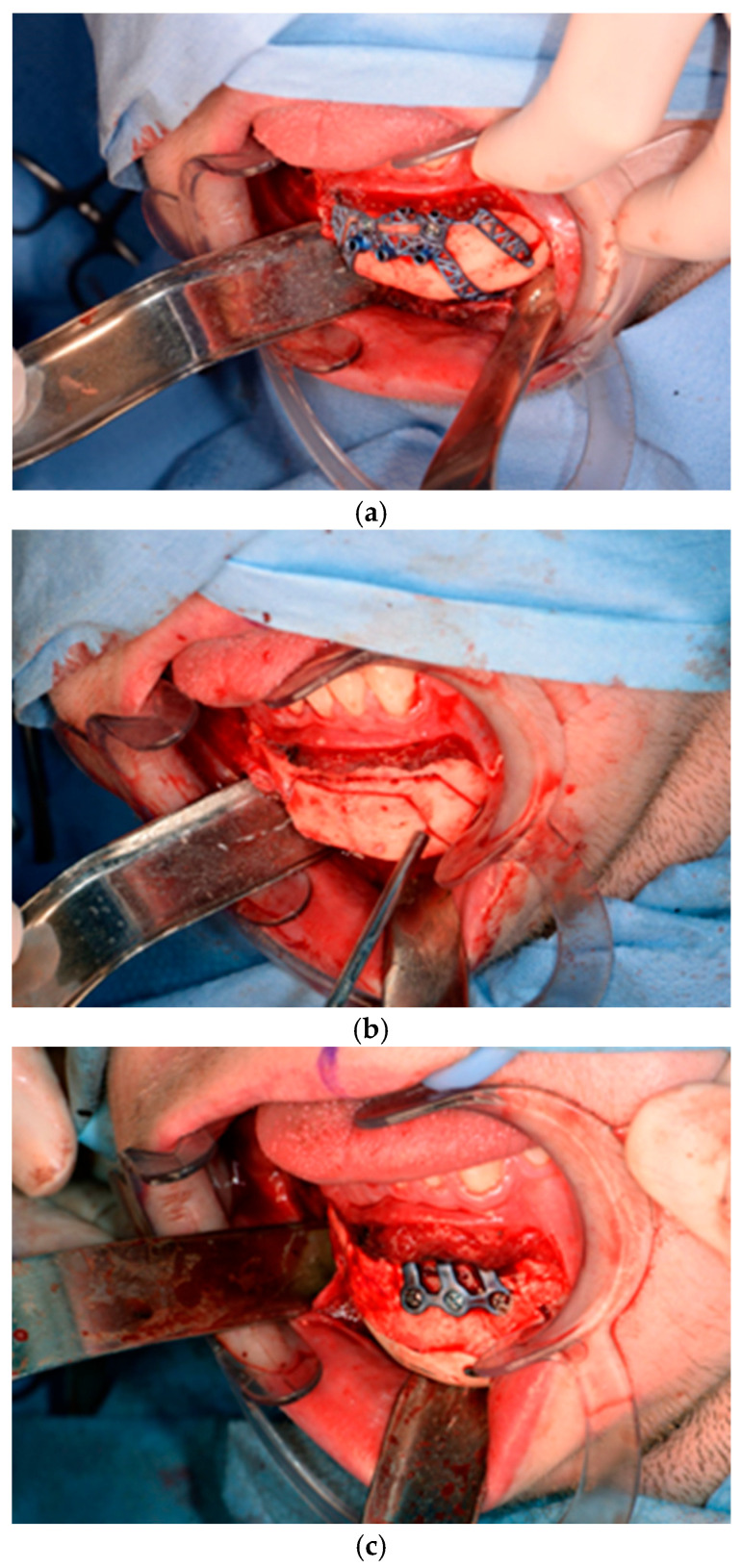
(**a**)—Placement of titanium cutting guide, (**b**)—genioplasty cuts demonstrated, (**c**)—centralising genioplasty fixed with patient-specific titanium plate and fit with patient-specific PEEK mandibular implant. Patient 30. Chin augmentation with the implant and 2 had mandibular PSI in combination with a centralising/rotation genioplasty using a titanium patient guide and patient-specific plate.

Of the five patients where the implant extended to the anterior mandible, two had direct chin augmentation with the implant and two had mandibular PSI in combination with a centralising/rotation genioplasty using a titanium patient guide and patient-specific plate [Figure 1a—Placement of titanium cutting guide. Figure 1b—Genioplasty cuts demonstrated. Figure 1c—Centralising genioplasty fixed with patient-specific titanium plate and fit with patient-specific PEEK mandibular implant. Patient 30].

Within this hemifacial microsomia subgroup, two patients had PSI secondary to orthognathic surgery and one a PSI as a primary camouflaging procedure. One patient received a three-part mandibular implant for mandible micrognathia secondary to juvenile idiopathic arthritis. The remaining patients received implants to camouflage post-traumatic deformities.

In 23 cases, the primary surgeon operated as the sole consultant, while in 33 cases, the surgery was performed jointly with another consultant. The orbital PEEK implant surgery was performed jointly between the maxillofacial surgeon and orbital ophthalmic surgeon. This collaboration enhanced decision-making and technique, potentially improving outcomes.

The use of virtual planning was used in 52 cases vs. 4 analogue in-house which further supports the precision and success of PEEK implants, allowing for more accurate implant placement and better surgical outcomes. The first PEEK implant was placed 28 June 2012. The first two PEEK implants placed were planned with virtual. The first analogue in-house was placed 23 August 2012 and the last was 17 July 2013. A total of 14 cases were planned with interlocking joints, with the first being placed 18 March 2015.

The surgical approaches used varied significantly, highlighting the adaptability of PEEK implants to different anatomical and clinical challenges. The approaches and times they were used were: 22 intraoral approach; 9 trans-conjunctival alone; 11 transconjunctival lid swing; 5 transconjunctival with lateral canthotomy; 3 transconjunctival with lateral canthotomy and retrocaruncular; 2 transconjunctival with retrocaruncular; 2 trans conjunctival lid swing and intra-oral; 1 mid-tarsal skin incision; and 1 bicoronal. Intra-oral access was used primarily for mandibular and zygomatic reconstructions. Transconjunctival approaches, either alone or combined with lateral canthotomy or retrocaruncular techniques, were predominantly used for orbital reconstructions. The diversity of surgical techniques employed demonstrates the versatility of PEEK implants in addressing different reconstructive needs and anatomical complexities.

The data on screws and implants reveal the level of customisation and precision involved in each procedure. The number of screws used ranged from 0 to 6, with the average number of screws used being 1.8. The average screw length was 7.29 mm. Screw length selection is needed for precise fixation without penetrating critical structures.

Additionally, implants were recorded to be soaked in antibiotics in 33 cases, predominantly with Gentamicin. Dosages included 40 mg dose in 9 cases, 80 mg dose in 3 cases, and 20 cases where no dose was recorded. A Clindamycin 80 mg dose was used once. This was for the first case. In the other cases, it was not recorded that the implant was soaked, but it is our practise to soak.

Preoperative planning scans were 50:50 split with 28 cases having a Cone Beam CT (CBCT) vs. 28 CT. Postoperative CT or CBCT scans were performed in 34 cases, facilitating the assessment of implant positioning and integration.

The mean follow-up period of 672 days provides a substantial timeframe for evaluating long-term outcomes.

## 4. Complications

**Figure 2:**
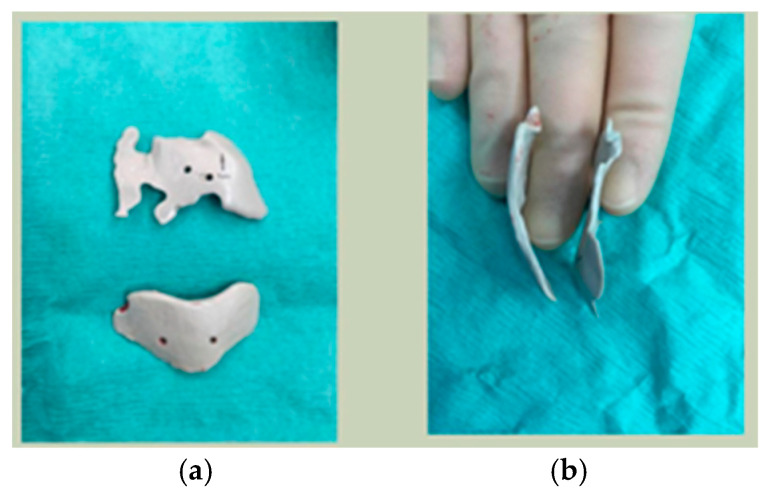
(**a**,**b**) Patient 7. The in-house planned zygoma implant that was removed placed next to the virtually planned implant prior to insertion.

**Figure 3:**
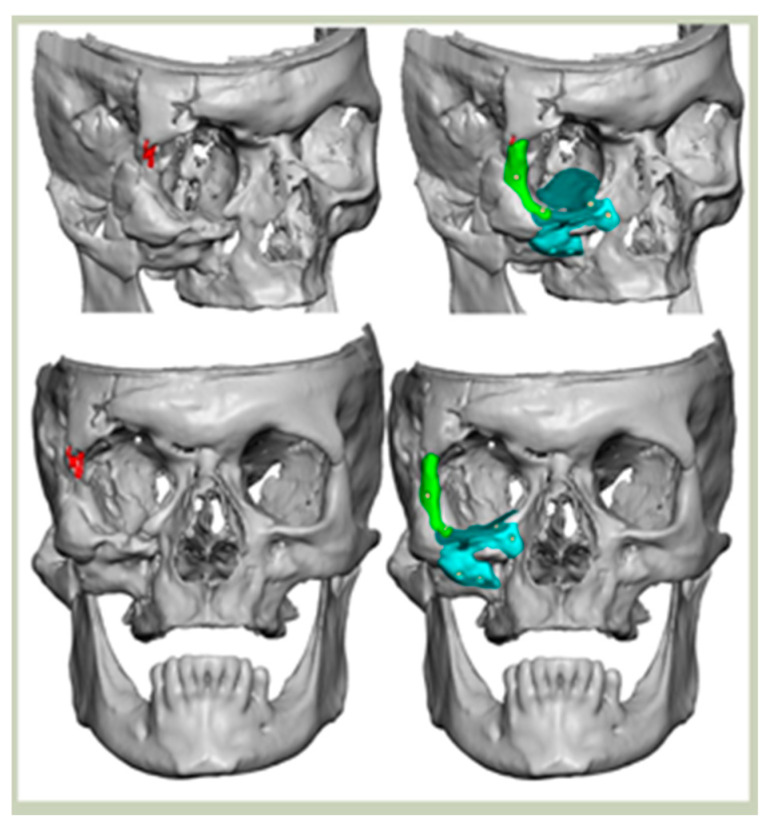
Patient 44. 3-part implant that extruded and was removed.

Most patients experienced no complications, highlighting the overall safety and effectiveness of PEEK implants. However, some complications were noted with 8.9% of cases resulting in swelling and/or infection. In 5.4% of cases, some paraesthesia was reported. Implants were removed in three patients. An in-house zygoma implant was replaced with a virtually planned implant as the first was too bulky [Figure 2a,b. Patient 7. The in-house planned zygoma implant that was removed was placed next to the virtually planned implant prior to insertion]. Another in-house zygoma implant had a poor fitting surface and became infected. The final one to be removed was a three-part orbital, zygoma and lateral wall virtually planned implant that extruded over the zygoma where the skin was very thin. This developed a chronic infection, which was found to be in part due to residual exogenous implant material, which we think were the remains of a previous silicone implant [Figure 3—Patient 44. Three-part implant that extruded and was removed].

**Figure 4:**
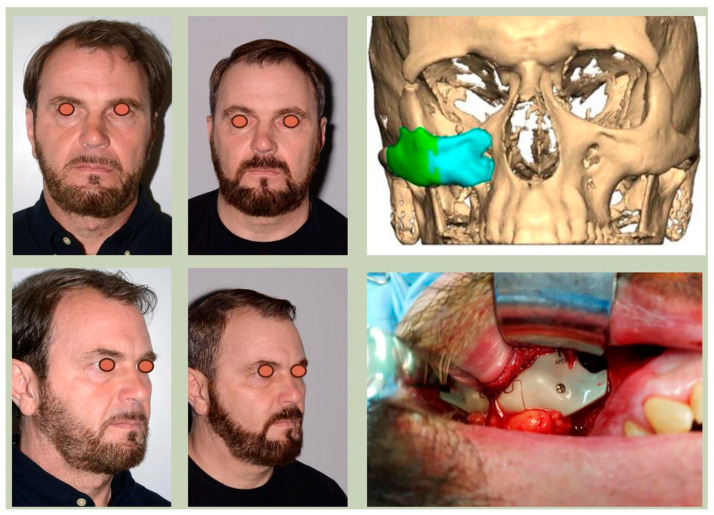
Patient. A right sided 2-piece zygomatic implant with an interlocking joint with maxillary sinus infection.

A total of 2 cases had recurrent sinusitis where screws penetrated into the sinus. A patient who received an interlocking two-part right zygomatic PEEK through an intra-oral approach, developed recurrent right maxillary sinus infection manifested by facial swelling but no signs of infection around the implant. A CT scan showed a wholly obliterated maxillary sinus and showed the fixation screw protruding into the sinus cavity. After referral to ENT, the patient was treated successfully with functional endoscopic sinus surgery (FESS), the implant still in situ and no complications three years post-FESS [Figure 4—Patient. A right sided two-piece zygomatic implant with an interlocking joint with maxillary sinus infection].

A patient with a three-part mandibular implant experienced bilateral altered sensation in the distribution of the mental nerve.

**Figure 5:**
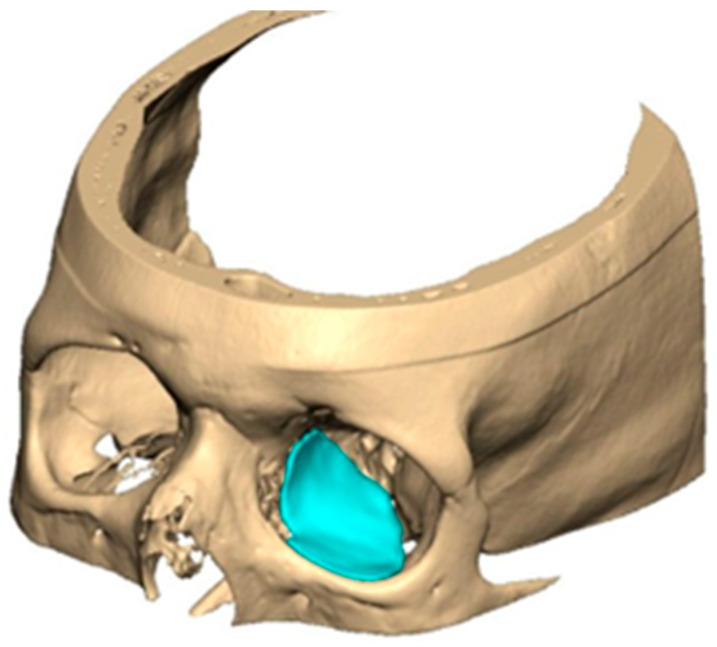
Patient 18. Implant not seated on posterior ledge. An improvement was still gained post-surgery. We would now plan with the implant sitting on the infra-orbital rim.

In 1 case, the implant was found to not have an ideal position on the posterior ledge, but there was still an improvement from preop. The patient was offered a replacement but declined [Figure 5—Patient 18. Implant not seated on posterior ledge. An improvement was still gained post-surgery. We would now plan with the implant sitting on the infra-orbital rim].

All patients had an uneventful discharge on the morning following their surgery. No interlocking implants showed signs of infection around them or required removal. The demographics, implant details, and complications for our cohort of patients are presented in Table 1.

## 5. Discussion

PEEK is a versatile material for craniofacial reconstruction, with increasing evidence supporting its use in the management of complex orbital and maxillofacial defects [7,8]. Reconstruction of the craniomaxillofacial region remains particularly challenging due to its intricate three-dimensional anatomy, characterised by variable curvatures and thicknesses [9]. Importantly, such reconstructions extend beyond aesthetic restoration, addressing functional impairment and the significant psychological consequences of craniofacial deformity.

The gold standard for bony reconstruction is autologous bone grafting. However, its widespread application is restricted by difficulties in intraoperative shaping, the risk of graft resorption, and donor site morbidity. To overcome these limitations, various alloplastic materials have been introduced, most notably, polyetheretherketone (PEEK), porous polyethylene (MEDPOR^®^), and titanium. Each of these materials offers specific advantages in restoring facial anatomy, with PEEK gaining prominence due to its favourable balance of strength, adaptability, and biocompatibility.

In contrast to titanium, pure PEEK exhibits one principal limitation: it is a hydrophobic material with no inherent bioactive properties and therefore does not actively bond to living tissue [10,11]. The long-term stability of PEEK implants is consequently dependent on secure screw fixation, precise three-dimensional design, and the accuracy of the implant–bone interface.

PEEK is increasingly utilised across multiple surgical disciplines, including maxillofacial surgery, orthopaedics, cardiovascular surgery, and neurosurgery. As an alloplastic material, it provides a favourable balance between structural strength and reduced rigidity, thereby lowering the risk of stress shielding when compared with more rigid alternatives such as titanium [12,13].

Our preference is to obtain a cone beam computed tomography (CBCT) scan prior to planning patient-specific implants. Brisco et al. demonstrated that CBCT delivers a significantly lower radiation dose compared with multislice CT, with the mean dose to the lens of the eye reduced by 42% (range 23–53%, SD 10; *p* < 0.001) [14]. Importantly, the effective dose—a measure of the risk of radiation-induced malignancy—was also significantly lower with CBCT than with multislice CT, making it the preferred modality for preoperative imaging in orbital reconstruction.

In our early experience, five PEEK implants were designed in-house and manufactured locally using an analogue workflow developed in Sheffield, which we term Digital–Analogue PEEK. This technique combined conventional modelling with digital scanning and CNC milling; wax patterns were fabricated on 3D-printed skull models, converted into acrylic, scanned, and then used to mill medical-grade PEEK implants. While cost-effective and reproducible, the method involved multiple manual steps and frequently required intraoperative adjustment to achieve an accurate fit. Limitations included occasional inaccuracies at the fitting surface due to cumulative errors in the pathway, the need for additional software correction of CBCT data in some cases, and a tendency for implants to be bulkier than their digitally designed counterparts.

By comparison, three-dimensional computer-assisted design and manufacturing (CAD/CAM) integrates virtual surgical planning (VSP) with direct CNC milling of PEEK, producing more precise and streamlined patient-specific implants. Thomas et al. demonstrated that CAD/CAM implants were significantly more accurate than those fabricated using in-house analogue methods [15].

During VSP the unaffected side is mirrored and used to restore damaged anatomy on the affected contralateral side. This new framework acts as a template on which a PSI is designed to onlay into sound bone. Screw holes are placed for implant fixation after checking the bone density and the maximum bone length so that screws are encased in bone. PSIs are designed following collaboration between surgeon and clinical engineer. CAD/CAM aids a more specific implant with more complex geometric shapes and the opportunity to link multiple implants with interlocking joints. During the design phase, consideration must be given to access required for placement, avoidance of critical anatomical structures, and soft tissue coverage.

Virtual PEEK Design Considerations

Implant Thickness

**Figure 6:**
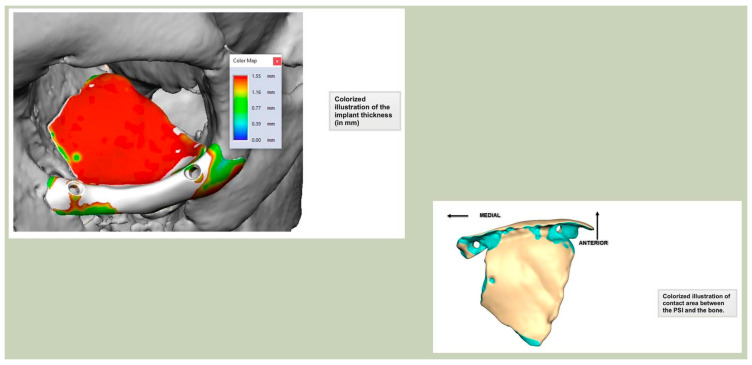
Thickness of an orbital implant and three points of contact.

For orbital reconstruction, PEEK implants were initially manufactured at 3 mm but have since been refined to 1.5 mm, providing adequate strength with reduced bulk. Cranial implants are generally supplied at 4 mm thickness, although 3 mm may be used in younger patients where clinically appropriate [Figure 6. Thickness of an orbital implant and three points of contact].

Seating and Fixation

**Figure 7:**
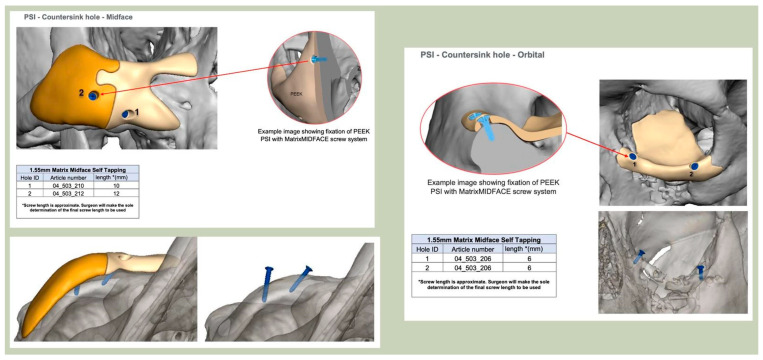
Planning to have fixation into sound bone.

Secure seating of the implant is essential. In zygomatic PEEK implants, the three-dimensional morphology of the anterior maxilla and zygoma often provides a natural seat, thereby improving stability. Although two screws are generally recommended to prevent rotation, in cases of firm seating a single screw may suffice. Implants are typically designed with an implant-to-bone offset of 0.2 mm to facilitate adaptation [Figure 7—Planning to have fixation into sound bone].

Positional Features

For relatively flat surfaces, such as the lateral orbital wall and the mandible, rims can be incorporated into the design to improve positioning. In orbital reconstruction, rims may also be extended across the infra-orbital margin to enhance stability. However, exposure of the infra-orbital rim risks scarring and lid malposition due to periosteal stripping. In our series, no such complications were observed. A single case of displacement occurred in the absence of infra-orbital rim coverage, despite intraoperative confirmation of positioning.

Orbital Plate Planning

Orbital plates are optimally designed with three points of contact along the orbital floor, ideally including one posterior point of fixation. An anterior ledge provides a useful visual reference for correct positioning during surgery. Since 2018, thinner plates (1.5 mm with tapered edges) have been routinely adopted, improving intraoperative handling and reducing bulk. Planned screw holes, incorporated since April 2019, have further enhanced accuracy and ease of fixation.

In our current practice, orbital implants are always designed with an extension over the infra-orbital rim. This improves stability and provides additional support to the lower eyelid following a transconjunctival approach, which often involves a lid swing. A ledge can also be incorporated in the infra-orbital region; however, this may create an unfavourable shadow and must be weighed against the morbidity of more extensive procedures such as zygomatic osteotomy.

**Figure 8:**
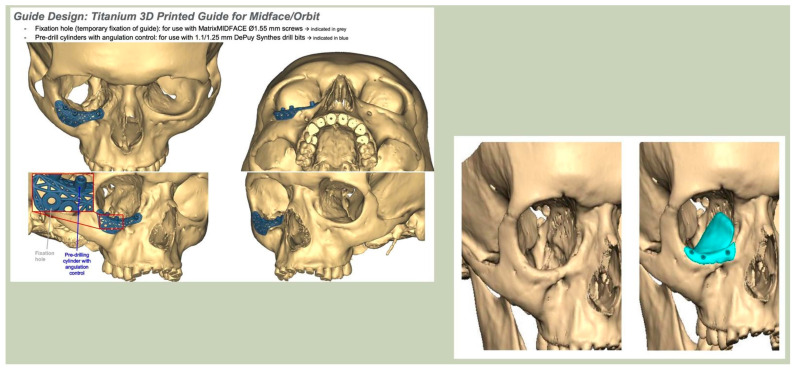
The use of a titanium drill guide to aid placement of a PEEK orbital implant.

With the advent of patient-specific drill/cutting guides and plates, orbital reconstruction has become increasingly predictable, with improved accuracy in implant placement [Figure 8—The use of a titanium drill guide to aid placement of a PEEK orbital implant].

PEEK-on-PEEK

**Figure 9:**
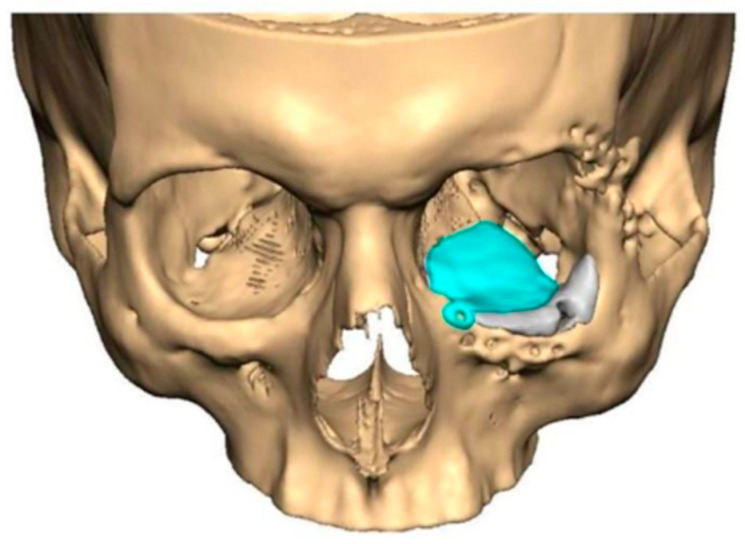
Patient 21—Image of patient-specific implant designed by the manufacturer. (DePuy Synthes)—Colourised illustration of PEEK-on-PEEK implant (max thickness 2.7 mm) show the original PEEK implant and the additional.

We have previously reported a case in which a patient underwent left orbital floor reconstruction with a patient-specific PEEK implant to correct globe dystopia. Despite the initial intervention, the patient developed progressive secondary enophthalmos and recurrent globe dystopia. To address this, a novel PEEK-on-PEEK technique was employed, whereby a second customised PEEK implant was placed directly over the original implant but within the same capsule, thereby augmenting orbital volume and improving globe position. This approach, termed PEEK-on-PEEK orbital implantation, represents a secondary augmentation strategy for the management of delayed enophthalmos [16] [Figure 9. Patient 21—Colourised illustration of PEEK-on-PEEK (max thickness 2.7 mm) showing the original PEEK implant (grey) and the additional implant (blue)].

Multi-piece Designs with Interlocking Joints

In selected cases, multi-piece PEEK implants can be produced, with segments linked together by interlocking joints. These are created during the digital design process using Boolean separation, followed by refinement to ensure accurate mating surfaces and stable intraoperative assembly. Interlocking designs provide additional stability, require minimal fixation, and facilitate ease of insertion [2,4,17].

**Figure 10:**
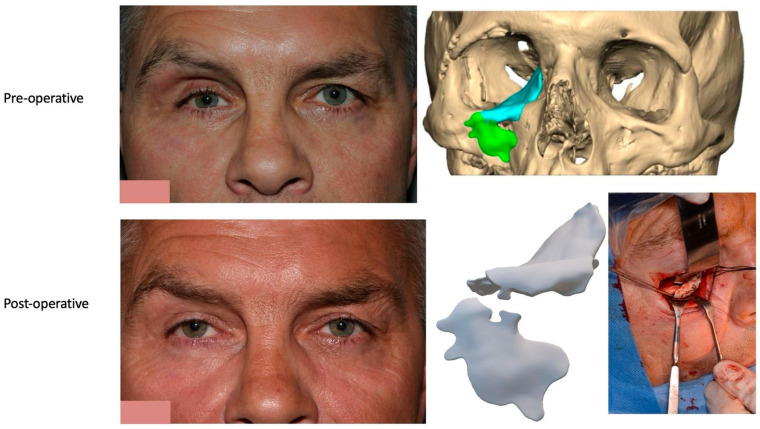
Patient 29. A two-part orbital-zygomatic implant linked with an interlocking joint.

Bespoke multi-segmented implants are particularly valuable when surgical access is restricted, when reconstruction of a large surface area is required, or when implants must be placed at different planes of the facial skeleton. Conventionally, each implant requires a minimum of two screws to prevent rotation; however, the use of interlocking joints reduces the number of screws required by effectively converting multiple segments into a single integrated implant [Figure 10—Patient 29. A two-part orbito-zygomatic implant linked with an interlocking joint].

Manufacturing

PEEK implants are manufactured using 5-axis computer numerical control (CNC) milling machines. Although the core milling process has remained largely unchanged, several refinements have been introduced following feasibility testing, including the addition of countersunk screw holes and plate recesses to improve handling and fixation. Earlier-generation implants were comparatively thicker and often required intraoperative trimming to achieve an accurate fit. The introduction of 1.5 mm orbital implants with tapered margins has markedly improved intraoperative adaptation, reduced the need for modification, and has been consistently associated with positive surgeon feedback.

Infection

Infection remains a key concern in cranioplasty and craniofacial reconstruction. In a two-centre cohort study of 40 patient-specific PEEK cranioplasty implants, Jonkergouw et al. reported an infection rate of 13% [18]. By comparison, Rosenthal et al. presented outcomes from 65 cases across three centres, with a lower infection rate of 7.7% [19]. A systematic review by Kwarcinski et al. compared infection rates across various implant materials, reporting an average rate of 7.9% for PEEK, which was comparable to 8.3% for titanium [20].

Biofilm formation on implant surfaces is a recognised risk factor for surgical site infection, which can lead to implant exposure, extrusion, and ultimately implant removal. Barkarmo et al. compared biofilm formation across different materials, including untreated PEEK, blasted PEEK, commercially pure titanium, and titanium alloy (Ti6Al4V) [21]. Their results demonstrated increased biofilm formation on blasted PEEK compared with the other groups. By contrast, as-prepared PEEK exhibited biofilm formation levels comparable with those of commercial titanium and titanium alloy. It is well established that surface roughness promotes bacterial adhesion and biofilm proliferation [22,23], which may account for the higher biofilm accumulation observed on blasted PEEK.

In our practice, implants are routinely soaked in Gentamicin (40–80 mg) prior to insertion, in line with local antimicrobial protocols.

Placement

PEEK also allows modification of the non-fitting surface of the implant with a bur to allow for symmetry and facial balance.

Although customised implants represent a high-cost product, the use of computer-aided design and manufacturing (CAD/CAM) has been shown to reduce both intraoperative manipulation and overall operative time [24]. CAD facilitates preoperative planning of implant position and the optimal length and orientation of fixation screws, while CAM enables the precise incorporation of screw holes directly into the implant design, thereby improving accuracy and efficiency at the time of surgery.

**Figure 11:**
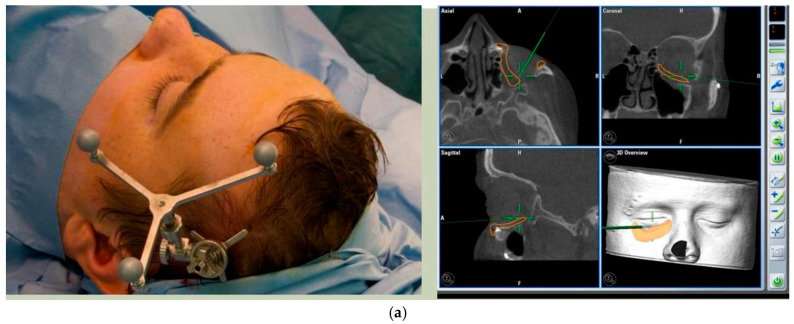

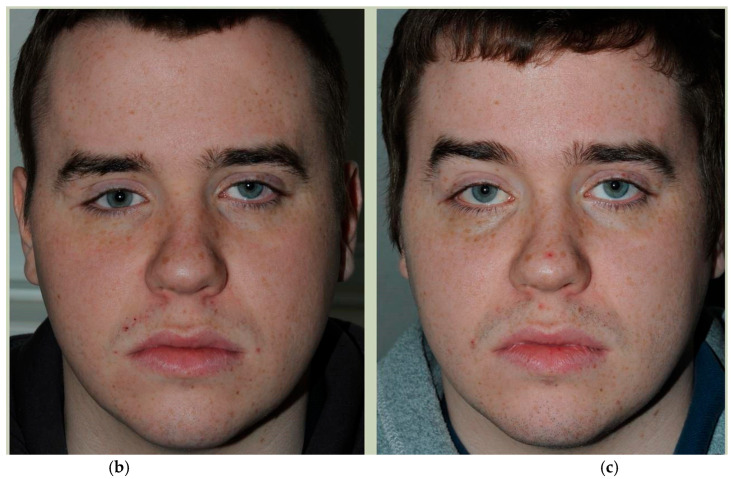
(**a**) Reference array of surgical navigation attached to skull and navigation used to confirm position of implant in planned position. (**b**) Preoperative photo showing globe dystopia. (**c**) Postoperative photo showing globe dystopia corrected. Patient 10. Right sided orbital implant placed with the aid of surgical navigation.

Prior to surgery, and if required, the implant can be placed in the planned position within the skull and imported as a segmented STL format file (Standard Tessellation Language) into our surgical navigation system (Brain Lab Navigation, Munich, Germany). Surgical navigation allows the surgeon to navigate around the craniomaxillofacial region and, once the implant has been surgically placed, navigation can be used to confirm satisfactory positioning of the implant by comparing the surgically achieved position to the planned position. Checking position can also be aided with the prior construction of a predesigned titanium patient-specific guide where fixation holes for the implant can be predrilled. For most of our PSIs, placed confirmation of planned position has been confirmed by the stability of the fitting surface of the implant with the underlying bony anatomy [Figure 11. Right sided orbital implant placed with the aid of surgical navigation].

If there are concerns on the extent of periosteal envelope achieved around the implant, especially in the region of the lower border of the mandible, including the angle of the mandible that is required to accommodate the implant, then multiple implants linked by interlocking joints can be constructed of different thicknesses at the lower border. This allows a safe thickness of the implant to be inserted within the envelope without the risk of future implant extrusion.

Over the decade of cases, our most challenging cases are post-trauma, which have had multiple attempts at reconstruction previously, often with other material used, compromising the bony framework that is used as the structure for the implant to sit on and the soft tissue envelope.

Clinical Outcomes of PEEK in Maxillofacial Surgery

Although the clinical use of PEEK in maxillofacial reconstruction has expanded considerably, there remains a limited body of high-level evidence. Current reports, however, suggest that PEEK implants are associated with favourable functional and aesthetic outcomes, combined with acceptable complication rates.

Gerbino et al. described 13 cases of post-ablative craniofacial reconstruction using patient-specific PEEK implants, of which 11 achieved satisfactory restoration of complex morphological defects with good cosmetic outcomes. Importantly, no implant-related complications were observed [1]. Järvinen et al. reported a retrospective cohort of 24 patients, in which PEEK implants fitted well without adjustment in 22 cases. Nine implants required modification of the outer contour, while two patients (8.3%) developed postoperative wound dehiscence and infection, both of which resolved without implant removal [25]. Similarly, Kim et al. presented four cases reconstructed with customised PEEK implants, all of which demonstrated excellent functional and aesthetic outcomes at 16–20 months of follow-up, without any complications such as infection or extrusion [26].

Longer-term evidence has also been published. Garg et al. reviewed 19 PEEK onlay implants placed over 11 years, primarily for orbital-zygomatic and mandibular reconstruction. Only two implants were removed—one for a design error and one due to compression neuropathy—while one case of postoperative infection was successfully managed with antibiotics [27]. Collier et al. reported on 50 consecutive cases of custom-made facial onlay implants over five years, of which 84% were manufactured from PEEK. Minor intraoperative adjustments were required in approximately one-third of cases, and a single patient required reoperation. Importantly, no implants were lost or removed during follow-up [28].

Infection remains a central concern in cranioplasty and craniofacial reconstruction. A two-centre study of 40 patient-specific PEEK cranioplasty implants reported an infection rate of 13% [18], while a multi-centre series of 65 cases demonstrated a lower rate of 7.7% [19]. A systematic review by Kwarcinski et al. found the average infection rate for PEEK to be 7.9%, comparable to titanium at 8.3% [20]. These findings suggest that PEEK carries a similar risk profile to other widely used materials. At the cellular level, Barkarmo et al. demonstrated that biofilm formation varies with implant surface preparation: blasted PEEK exhibited higher bacterial adhesion, while untreated PEEK showed levels comparable with commercial titanium and titanium alloy [21]. As surface roughness is known to promote bacterial adherence [22,23], this may partially explain variations in reported infection outcomes.

Taken together, these studies suggest that PEEK implants offer satisfactory long-term stability, with low complication and removal rates, and outcomes at least comparable to established alternatives such as titanium. However, the literature is characterised by retrospective cohorts with small sample sizes and limited follow-up, underlining the need for prospective multi-centre studies to establish the long-term safety and efficacy of PEEK in maxillofacial reconstruction.

We have previously described the use of interlocking joints to connect multiple PEEK implants, providing more controlled and predictable placement in cases where the implant lacks a straightforward path of insertion, involves multiple planes, or must be positioned around vulnerable anatomical structures such as the mental or infra-orbital nerves. Smaller implant segments that lock together within the periosteal envelope also facilitate insertion through limited surgical access with reduced periosteal stripping. However, it remains essential that the periosteal pocket is of sufficient size to accommodate the final assembled implant without creating undue soft tissue tension.

Implant stability is paramount to reducing the risks of infection, migration, and exposure. Even with an accurately fitting surface, fixation with at least two screws is recommended to prevent rotational movement. The use of a three-part interlocking PEEK patient-specific implant (PSI) allows the completed construct to be stabilised and secured with just two screws, reducing the overall fixation requirements. Looking forward, the integration of soft tissue simulation during preoperative planning may further refine implant design, enabling adjustments for the quality and thickness of overlying soft tissues to optimise both stability and aesthetic outcomes.

**Figure 12:**
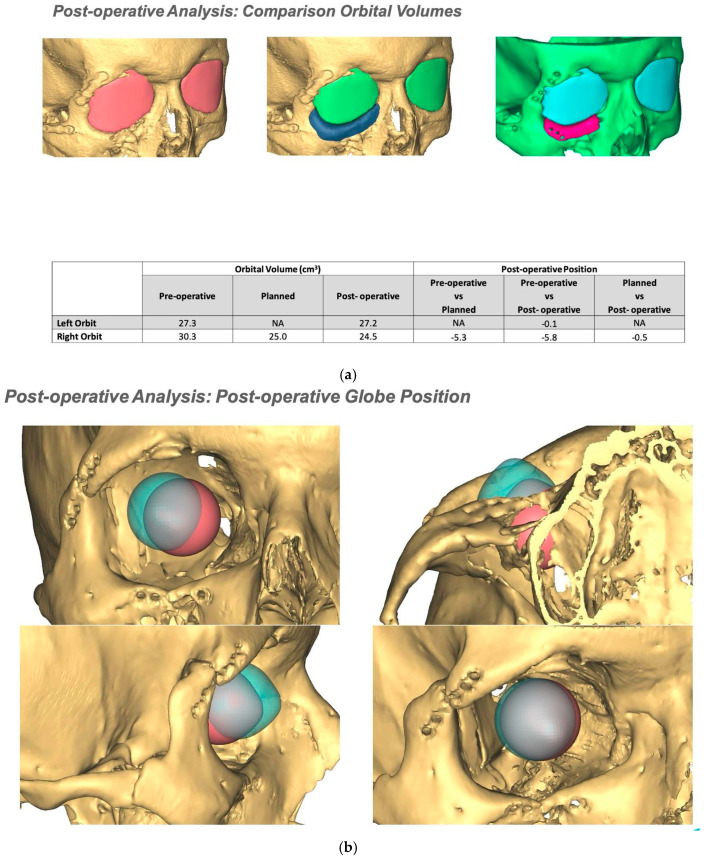

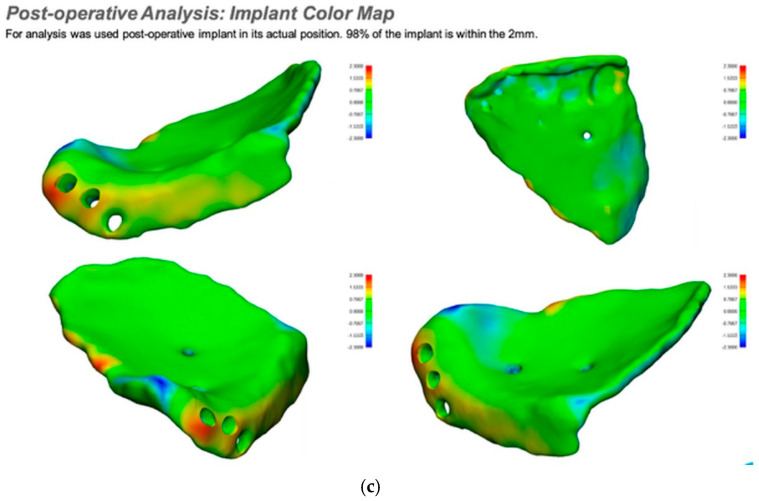
Post planning reports looking at (**a**) orbital volume, (**b**) globe position correction, and (**c**) the ability to check if the achieved implant position matched the planned implant position.

If required and a post-surgical CT scan is available a post-surgical planning report can be undertaken. This is of particular use in the reconstruction of orbital injuries where the correction of globe dystopia and orbital volume can be analysed. It is also possible to assess how close the achieved implant position was to the planned implant position [Figure 12—Post planning reports looking at (a) orbital volume, (b) globe position correction, and (c) the ability to check if the achieved implant position matched the planned implant position].

## 6. Conclusions

The use of patient-specific PEEK implants (PSIs) is safe, predictable, and can reduce operative time while allowing intraoperative adjustability. Virtual surgical planning enables the consistent production of customised implants, incorporating features such as reduced thickness, precisely positioned screw holes, and confirmation that fixation points engage sound bone. Unlike titanium, PEEK can be modified intraoperatively if required, although the fitting surface should not be altered. Virtually planned PEEK implants can also be integrated with other forms of personalised surgery, including patient-specific orthognathic guides and plates, to achieve comprehensive reconstruction.

The incorporation of an interlocking mechanism provides additional stability, prevents implant slippage, and reduces the need for supplementary fixation—particularly valuable in anatomically inaccessible regions or in proximity to vital structures. Multi-segmented designs also facilitate insertion through smaller surgical access incisions, without compromising functional or aesthetic outcomes. Furthermore, intraoperative navigation can be used to improve accuracy of implant placement, with post-planning reports generated to confirm that the implant has been positioned in accordance with the virtual plan.

A multi-disciplinary approach, such as dedicated joint orbital trauma clinics and joint operating by maxillofacial surgery teams and orbital ophthalmic teams. helped improve patient outcomes in complex orbital PEEK implant cases.

## Figures and Tables

**Table 1:** Cohort demographics and results.

Patient	Sex and Age at Time of Surgery (yr)	Indication	Smoking Status	Image	Side	Site	Approach	Number of Pieces	Screws	Follow-Up Since Surgery (Days)	Complications
1	39M	Trauma	No	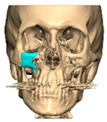	Right	Zygoma	Intra-oral	1	1	607	Right soft tissue swelling
2	41M	Trauma	No	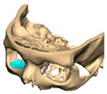	Right	Orbital	Transconjunctival	1	2	121	None
3	42F	Trauma	Smoker	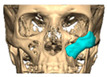	Left	Zygoma	Intra-oral	1	2	26	None
4	20M	Trauma	Smoker	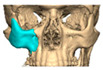	Right	Zygoma	Intra-oral	1	1	489	None
5	47M	Trauma	No	In-house planning. No scan image	Left	Orbital	Transconjunctival	1	0	134	Needed squint surgery
6	33M	Trauma	Smoker	In-house planning. No scan image	Right	Orbital	Mid-tarsal skin incision	1	2	147	None
7a	31M	Trauma	Smoker	In-house planning. No scan image	Left	Zygoma	Intra-oral	1	1	881	Implant was removed as it was more prominent
8	32M	Trauma	Smoker	In-house planning. No scan image	Left	Zygoma	Intra-oral	1	1	No follow up recorded. Pt DNA	None
9	23M	Trauma	No	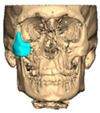	Right	Zygoma	Intra-oral	1	1	3614	Sinus swelling and inflammation around the screw
10	22M	Trauma	No	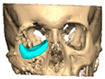	Right	Orbital	Transconjunctival lid swing	1	2	398	None
11	32M	Trauma	Smoker	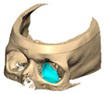	Left	Orbital	Transconjunctival lid swing	1	2	149	None
7b	33M	Trauma	Smoker	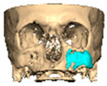	Left	Zygoma	Intra-oral	1	2	12	None
12	44M	Trauma	Smoker	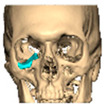	Right	Orbital	Transconjunctival lid swing	1	1	44	None
13	20M	Deformity	No	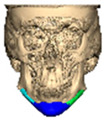	Bi lateral	Mandible	Intra-oral	3	2	1834	None
14a	17F	Deformity	No	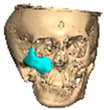	Right	Zygoma	Intra-oral	1	2	2463	None
15	50M	Trauma	No	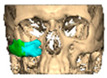	Right	Zygoma	Intra-oral	2	1	1886	Swelling right cheek treated with antibiotics
16	31F	Trauma	No	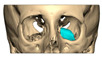	Right	Orbital	Transconjunctival	1	1	568	None
17	29M	Trauma	Smoker	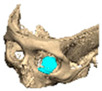	Left	Orbital	Transconjunctival lid swing	1	1	1154	None
14b	17F	Deformity	No	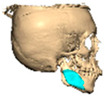	Right	Mandible	Intra-oral	2	1	2253	None
18	66F	Trauma	No	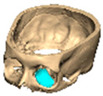	Left	Orbital	Transconjunctival	1	0	421	Posterior displacement into the sinus
19	51F	Trauma	Smoker	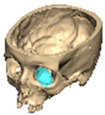	Left	Orbital	Transconjunctival, lateral canthotomy, and transcaruncular	1	1	50	Contacted with intermittent redness around left orbit and tender lump inferior rim. Treated with antibiotics.
20	28M	Deformity	No	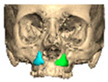	Left	Anterior Maxilla	Intra-oral	2	2	173	None
21a	30M	Trauma	No	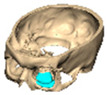	Left	Orbital	Transconjunctival and lateral canthotomy	1	1	2598	Developed further enophthalmos. New onlay PEEK placed
22	72F	Trauma	Smoker	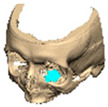	Left	Orbital	Transconjuntival	1	1	421	None
23	38M	Trauma	No	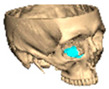	Right	Orbital	Transcaruncular and transconjunctival	1	1	330	None
24	42M	Trauma	No	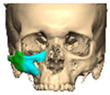	Right	Zygoma	Intra-oral	2	2	209	None
25	19M	Trauma	Smoker	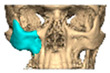	Right	Orbital	Transconjunctival	1	1	1038	None
26	46F	Trauma	Smoker	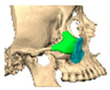	Right	Zygoma	Intra-oral	2	1	265	None
27	50M	Trauma	Smoker	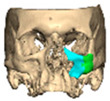	Left	Zygoma	Intra-oral	2	2	139	None
28	49M	Trauma	No	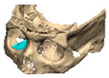	Right	Orbital	Transconjunctival, lateral canthotomy, and transcaruncular	1	1	1413	Screw does penetrate into the sinus. Had squint surgery and lower lid tightening procedure
29	53M	Trauma	No	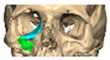	Right	Orbital and Zygoma	Transconjunctival, lid swing, and intra-oral	2	1	477	None
30	20M	Deformity	No	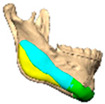	Right	Mandible	Intra-oral	2	3	1749	None
31	30F	Trauma	No	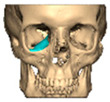	Right	Orbital	Transconjunctival	1	2	1198	None
32	20F	Deformity	No	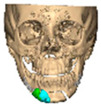	Right	Mandible	Intra-oral	2	2	1630	None
33	44M	Trauma	No	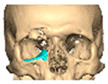	Right	Orbital	Transconjunctival, lateral canthotomy, and transcaruncular	1	2	754	None
34	24F	Deformity	No	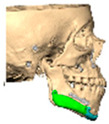	Bi lateral	Mandible	Intra-oral (Labial incision LR8-LL3)	2	3	201	Numbness lower lip
35	35M	Deformity	No	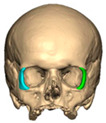	Bi lateral	Lateral Orbits (Bilateral)	Bicoronal	1	3 left 2 Right	603	None
36	18M	Trauma	No	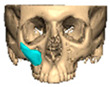	Right	Zygoma	Intra-oral	1	2	195	None
37	23M	Trauma	No	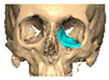	Left	Orbital	Transconjunctival and lateral canthotomy	1	2	435	Sudden onset of pain and paraesthesia
38	45F	Trauma	No	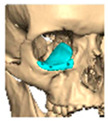	Right	Orbital	Transconjunctival	1	3	988	Needed strabismus surgery for diplopia. Complications with inferior rectus muscle
39	27M	Trauma	Smoker	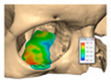	Right	Orbital	Transconjunctival and lid swing	1	2	694	None
40	60M	Trauma	No	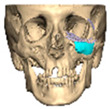	Left	Zygoma	Intra-oral	1	2	163	Slight altered sensation
41	52F	Trauma	No	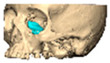	Left	Orbital	Transconjunctival and transcaruncular	1	2	162	None
42	25M	Trauma	No	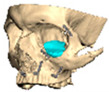	Left	Orbital	Transconjunctival and lateral canthotomy	1	2	715	None
43	40M	Trauma	No	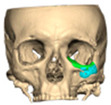	Left	Orbital and Zygoma	Transconjunctival and lid swing	2	4	792	Squint surgery
44	73M	Trauma	No	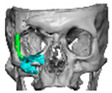	Right	Zygoma, lateral wall of orbit and orbital floor	Transconjunctival and lid swing and intra-oral	3	6	883	Needed implant removal following extrusion and then infection, which required further surgery
45	67F	Trauma	No	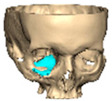	Right	Orbital	Transconjunctival and lateral canthotomy	1	2	491	Pain
46	61F	Trauma	No	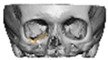	Right	Orbital	Transconjunctival and lid swing	1	2	295	Diplopia
47	23M	Trauma	No	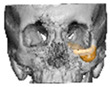	Left	Orbital and Zygoma	Transconjunctival and lid swing	2	4	148	None
48	20M	Trauma	Smoker	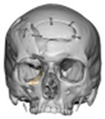	Right	Orbital	Transconjunctival and lateral canthotomy	1	2	36	None
49	29M	Deformity	No	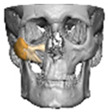	Right	Zygoma	Intra-oral	2	2	122	None
50	38F	Trauma	Smoker	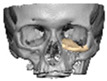	Left	Orbital	Transconjunctival and lid swing	1	2	193	Sinusitis
21b	37M	Trauma	No	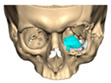	Left	Orbital	Transconjunctival and lid swing	1	1	132	None
51	38M	Trauma	No	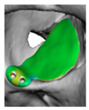	Right	Orbital	Transconjunctival	1	2	29	None
52	23F	Deformity	No	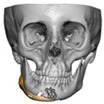	Right	Mandible	Intra-oral	2	2	48	None
53	41M	Trauma	Smoker	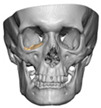	Right	Orbital	Transconjunctival and lid swing	1	2	15	None

## Data Availability

The original contributions presented in this study are included in the article. Further inquiries can be directed to the corresponding author.
